# Anti-Quorum Sensing Activity of Stevia Extract, Stevioside, Rebaudioside A and Their Aglycon Steviol

**DOI:** 10.3390/molecules25225480

**Published:** 2020-11-23

**Authors:** Victor Markus, Orr Share, Kerem Teralı, Nazmi Ozer, Robert S. Marks, Ariel Kushmaro, Karina Golberg

**Affiliations:** 1 Department of Medical Biochemistry, Faculty of Medicine, Near East University, Nicosia 99138, Cyprus; victor.markus@neu.edu.tr (V.M.); kerem.terali@neu.edu.tr (K.T.); 2 Avram and Stella Goldstein-Goren Department of Biotechnology Engineering, Ben-Gurion University of the Negev, Be’er Sheva 84105, Israel; orrs@post.bgu.ac.il (O.S.); rsmarks@bgu.ac.il (R.S.M.); 3 Department of Biochemistry, Faculty of Pharmacy, Girne American University, Kyrenia 99428, Cyprus; nazmiozer@gau.edu.tr; 4 The Ilse Katz Center for Meso and Nanoscale Science and Technology, Ben-Gurion University of the Negev, Be’er Sheva 84105, Israel

**Keywords:** quorum sensing, inhibition, stevia extract, stevioside, rebaudioside A, steviol

## Abstract

Governments are creating regulations for consumers to reduce their sugar intake, prompting companies to increase the ratio of artificial sweeteners in their products. However, there is evidence of some deleterious effects ascribed to the aforementioned synthetic agents and therefore consumers and food manufacturers have turned their attention to natural dietary sweeteners, such as stevia, to meet their sweetening needs. Stevia is generally considered safe; however, emerging scientific evidence has implicated the agent in gut microbial imbalance. In general, regulation of microbial behavior is known to depend highly on signaling molecules via quorum sensing (QS) pathways. This is also true for the gut microbial community. We, therefore, evaluated the possible role of these stevia-based natural sweeteners on this bacterial communication pathway. The use of a commercial stevia herbal supplement resulted in an inhibitory effect on bacterial communication, with no observable bactericidal effect. Purified stevia extracts, including stevioside, rebaudioside A (Reb A), and steviol revealed a molecular interaction, and possible interruption of Gram-negative bacterial communication, via either the LasR or RhlR receptor. Our in-silico analyses suggest a competitive-type inhibitory role for steviol, while Reb A and stevioside are likely to inhibit LasR-mediated QS in a non-competitive manner. These results suggest the need for further safety studies on the agents.

## 1. Introduction

*Stevia rebaudiana* Bertoni is a shrublike plant widely used as a sugar replacement in beverages and foods. The plant originated in South America, where for centuries it was used by indigenous people as a hypoglycemic agent and sweetener in drinks [1]. In the early 1930s, stevioside, the principal steviol glycoside responsible for the characteristic sweet taste in the stevia leaves, was first isolated [2]. Additional steviol glycosides, such as dulcoside A (Dulc A), Reb A, B, C, D, and E, were later isolated from stevia leaves. The predominant glycosides in stevia leaves from a dry-weight basis include stevioside (~9.1%), Reb A (~2.3%), Reb C (~0.6%), and Dulc A (~0.3%) [2]. Stevioside and Reb A, the two most abundant steviol glycosides, vary from each other by only one glucose moiety, where stevioside contains two glucose molecules and Reb A contains three [1]. Most of the studies on steviol glycosides were carried out on the two prominent members, stevioside and Reb A, molecules that are converted to their aglycon steviol in the colon [3,4,5]. Other non-prominent molecular members, such as steviolbioside, Dulc A, Reb B, C, D, E, F, and M, were found to share the same metabolic fate [6].

The hypoglycemic properties of steviol glycosides [7,8] may enhance insulin release and utilization [7,9,10], while aglycon steviol was shown to induce insulin secretion by directly acting on β-cells [11,12]. However, some studies could not ascertain the action of stevia in glucose regulation, nor could they find evidence that it could rescue mice with glucose intolerance, induced by a high-fat diet. Furthermore, administration of stevia resulted in similar disruption of the gut microbiota, as observed with saccharin [13]. The administration of stevia together with an obesogenic diet disrupted the gut microbiota, metabolism, and mesolimbic reward system in rat dams and their new-born [14]. Reb A interferes with gut microbiota balance and reduces dopamine transporter mRNA and nucleus accumbens tyrosine hydroxylase levels relative to the control [15]. Although some reports have noted that the steviol glycosides posed little pressure on gut microbial composition and growth [16,17], alteration of gut microbial balance by stevia glycosides is not in doubt [14,15]. Yet the mechanism by which stevia extract impacts the gut microbiota remains poorly understood.

In the gut microbiota as in many environments, QS is an essential and pivotal communication system that enables many aspects of bacterial community behavior to be regulated [18]. QS enables same or different species to interact and adjust their gene expression collectively based on their population density, either for the benefit of the whole population or for the competitive advantage of one species over another [19,20,21,22]. QS has been studied extensively in Gram-negative and Gram-positive pathogenic and symbiotic bacteria, [23,24,25,26]. Interestingly, this communication system is not just confined to bacteria but can be part of two-way cell-cell signaling including eukaryotic cells (both uni- and multicellular) [19,20,21,22]. These signaling molecules of QS are commonly referred to as autoinducers (AIs). In Gram-positive bacteria, AIs are either unmodified peptides or post-translational modified ones [27], with differences found in their structure and sequence [28,29,30]. In Gram-negative bacteria, the primary AIs are known as *N*-acyl homoserine lactones (AHLs) [31,32]. These signaling molecules are the most common class of AIs and are permeable through the membrane where they bind to their cognate receptor LuxR homologs in the cytoplasm and exert regulatory functions [30]. Different bacterial species may produce different AHLs, which vary in the length and substitution of the acyl chain but contain the same homoserine lactone moiety [33]. AHLs are produced via the LuxI protein family proportional to bacterial density until the AHLs reach a concentration level, or threshold, that form a LuxR–AHL complex, which then acts as a transcriptional factor to regulate a target gene [34,35]. Emerging evidence from the human gut reveal a connection between the gut microbiota homeostasis and the presence of a novel AHL signaling molecule (3-oxo-C12:2-HSL) [36,37,38], and other categories such as autoinducer-2 (AI-2) and autoinducer-3 (AI-3). AI-2 was proposed as a “universal” signaling molecule, for interspecies communication [24]. The molecular structure of AI-3 responsible for the pathogenesis of enterohemorrhagic *Escherichia coli* (EHEC), has just been elucidated [39].

In the present study, we evaluated the effect of stevia extract, steviol glycosides (stevioside and Reb A), and their derived aglycon steviol, on the AHL-dependent bacterial communication system.

## 2. Results

### 2.1. Anti-QS Activity of Commercial Stevia Herbal Supplement (CSHS)

**Figure 1:**
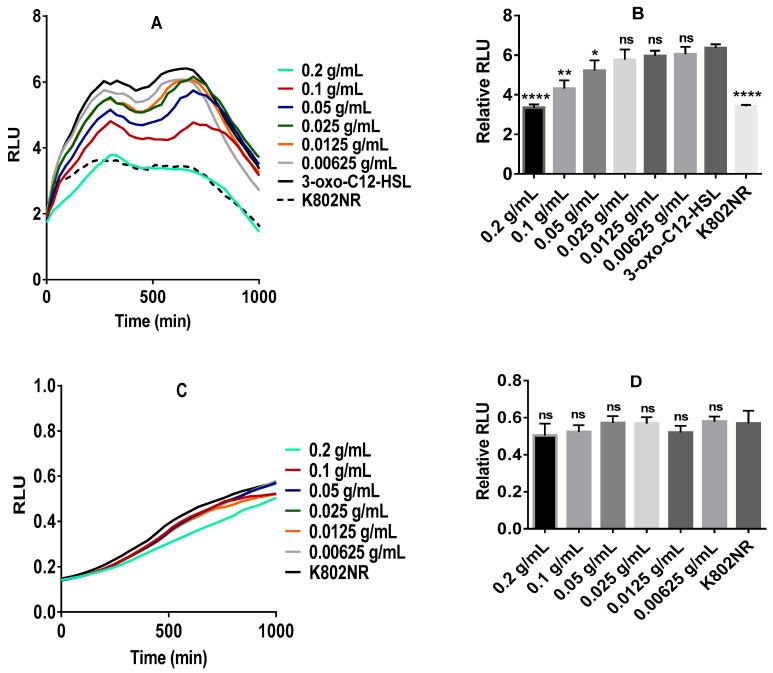
Anti-QS activity of CSHS. (**A**) Inhibitory effect of CSHS on the bioluminescence emission of K802NR reporter strain; (**B**) inhibitory effect of CSHS on K802NR bioluminescence emission relative to the control, consistent with the data in panel A; (**C**) growth response of K802NR reporter strain in the presence of CSHS; (**D**) growth response of K802NR in the presence of CSHS relative to the control, consistent with the data in panel C. Error bars are not shown in A and C to ensure clarity of figures. The final concentration of 3-oxo-C_12_-HSL was 5 × 10^−10^ M. All presented concentrations are the final concentrations. The amount of luminescence is expressed in relative light units or RLUs. * *p* < 0.05, ** *p* < 0.01, **** *p* < 0.0001, and ns not significant. Values represent mean ± SD, *n* = 3 (three different experimental readings).

To assess the potential effect of stevia on the QS system, we began with commercial stevia herbal supplement (CSHS). The product was purchased and tested against the recombinant bioluminescent *E. coli* K802NR-pSB1075 [40]. In the absence of *N*-(3-oxododecanoyl)-l-homoserine lactone (3-oxo-C12-HSL), only residual low light was produced. The addition of 3-oxo-C12-HSL to K802NR induced intense light production. The 3-oxo-C12-HSL molecule binds and activates the cytosolic receptor LasR that drives the transcription of target genes [18] including bioluminescence genes. Introduction of CSHS markedly decreases the K802NR bioluminescent emission (Figure 1A), apparently by interfering with the binding of 3-oxo-C12-HSL to the cytosolic receptor LasR. Importantly, CSHS appears not to have a bactericidal effect (Figure 1C).

### 2.2. LC-MS Analysis of CSHS

**Figure 2:**
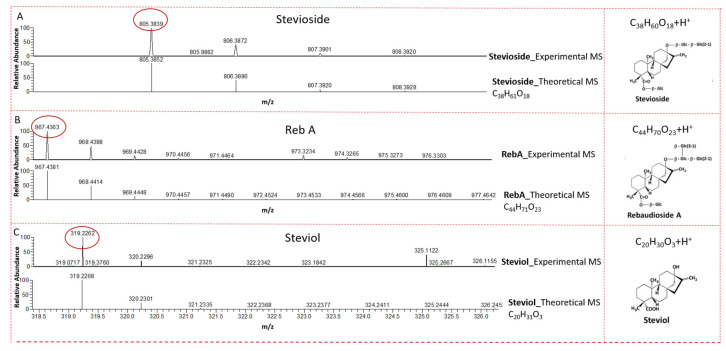
Experimental and theoretical mass spectrum of CSHS using ion trap MS in positive ion mode. (**A**) Stevioside; (**B**) Reb A; (**C**) steviol. On the right are the structures of the glycosides, along with their molecular formulas.

To ascertain the presence of the steviol glycosides in the CSHS, we performed an LC-MS analysis [41]. Previous studies have shown that stevioside and Reb A, derivative glycosides of steviol, are the two most abundant glycosides in stevia [1,2]. As expected, our result showed the presence of the prominent compounds, stevioside and Reb A, and their derived aglycon steviol in the supplement (Figure 2 and Figure S1). The characteristic fingerprint of the fragments of these compounds of interest, which agree with what has been reported in literature and MS libraries, are shown in Supplementary Figures S2–S4.

### 2.3. Anti-QS Activity of Pure Stevia-Derived Components

**Figure 3:**
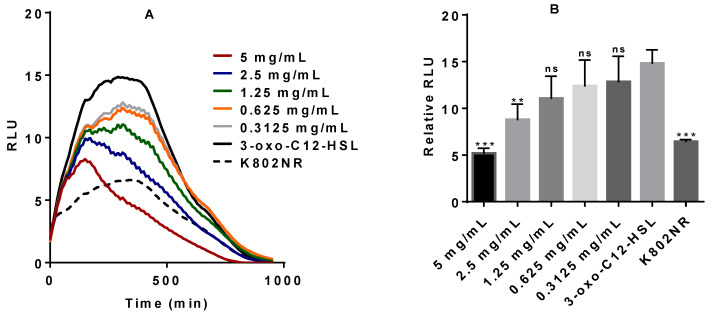

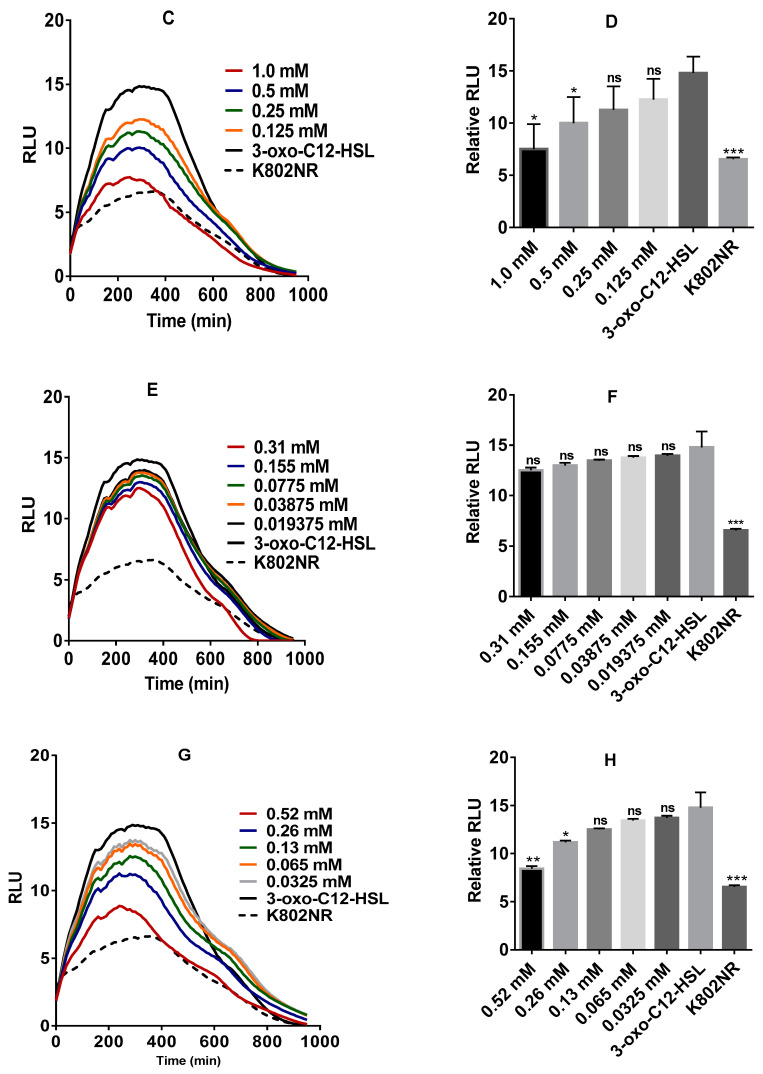
The bioluminescent response of K802NR reporter strain in the presence of pure stevia-derived components. (**A**) Stevia extract; (**B**) stevia extract relative to the control, corresponding to the data in panel A; (**C**) stevioside; (**D**) stevioside relative to the control, corresponding to the data in panel C; (**E**) Reb A; (**F**) Reb A relative to the control, corresponding to the data in panel E (**G**); steviol; (**H**) steviol relative to control, corresponding to the data in panel G. Error bars are not shown in A, C, E, and G to ensure clarity of figures. The final concentration of 3-oxo-C_12_-HSL was 5 × 10^−10^ M. All presented concentrations are the final concentrations. The amount of luminescence is expressed in relative light units or RLUs. * *p* < 0.05, ** *p* < 0.01, *** *p* < 0.001, and ns not significant. Values represent mean ± SD, *n* = 3 (three different experimental readings).

To further ascertain the anti-QS activity of the steviol glycosides, different concentrations of pure stevia extracts, stevioside and Reb A, as well as their derived aglycon steviol, were tested on the K802NR reporter strain. With the exception of Reb A, the results show an efficient inhibitory effect, as observed by a decrease in the bioluminescent emission, as shown in Figure 3.

**Figure 4:**
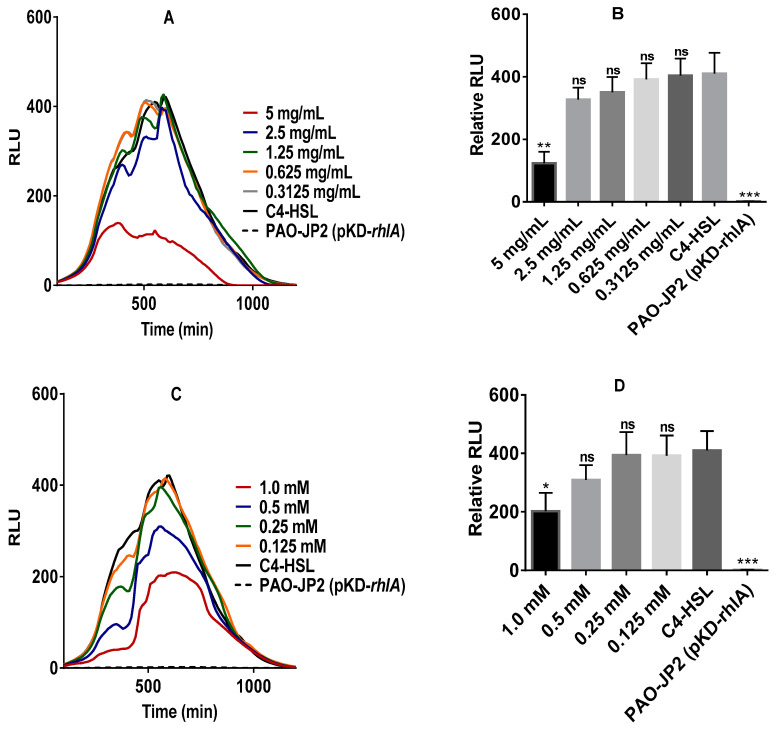

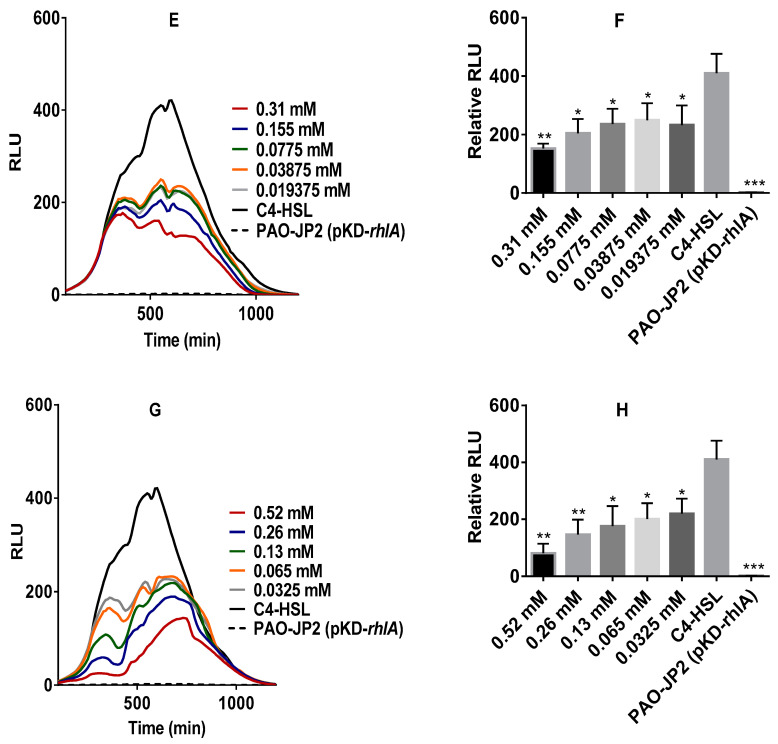
Inhibition of bioluminescence emission of PAO-JP2 (pKD-*rhlA*) by pure stevia-derived components. (**A**) stevia extract; (**B**) stevia extract relative to the control, correlating to the data in panel A; (**C**) stevioside, (**D**) stevioside relative to the control, correlating to the data in panel C; (**E**) Reb A, (**F**) Reb A relative to the control, correlating to the data in panel E; (**G**) steviol; (**H**) steviol relative to control, correlating to the data in panel G. Error bars are not shown in A, C, E, and G to ensure clarity of figures. The final concentration of C4-HSL was 10 µM. All presented concentrations are the final concentrations. The amount of luminescence is expressed in relative light units or RLUs. * *p* < 0.05, ** *p* < 0.01, *** *p* < 0.001, and ns not significant. Values represent mean ± SD, *n* = 3 (three different experimental readings).

When the PAO-JP2 (pKD-*rhlA*) strain [42] was exposed to all the stevia components, all had an efficient inhibitory effect on the bioluminescence emission of the bioreporter (Figure 4). While the stevia extract and stevioside showed significant inhibitory activity at the highest concentration, Reb A and steviol showed significant inhibitory activity across all the concentrations tested. In PAO-JP2 (pKD-*rhlA*), C4-HSL binds to RhlR protein to form the regulatory complex that modulates the transcription of target genes including *luxCDABE* [43], the process which was apparently disrupted by the stevia components.

### 2.4. In-Silico Studies

**Figure 5:**
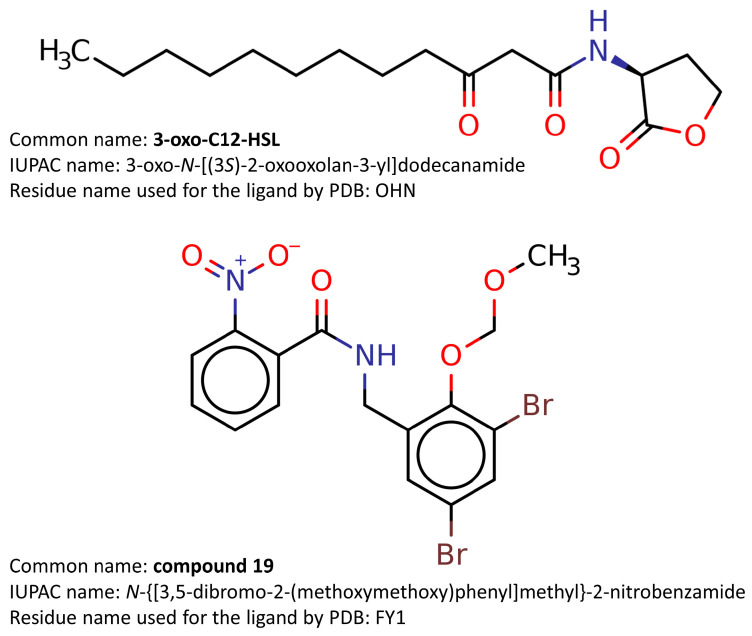
Molecular structures of the bona fide LasR ligands 3-oxo-C12-HSL (native autoinducer) and compound **19** (non-native agonist).

In an attempt to test the ability of our docking methodology to correctly recognize the ligand-binding cavity of LasR and faithfully reproduce the crystallographically determined LasR–ligand complexes, we performed redocking studies with the bona fide LasR ligands 3-oxo-C12-HSL (native autoinducer) and compound **19** (non-native agonist) (Figure 5).

**Figure 6:**
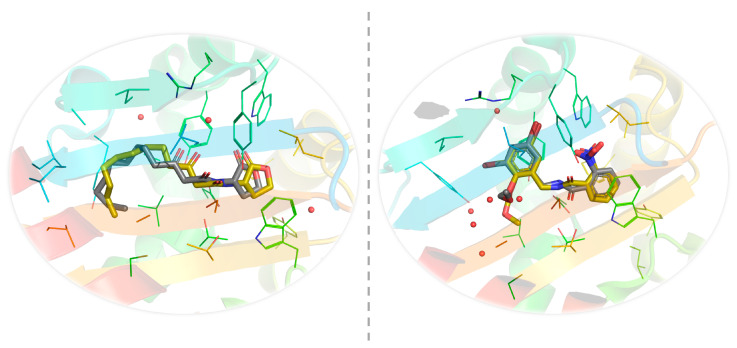
Results of the redocking calculations. **Left panel**: close-up view of the superposed structures of native (gold) and predicted (silver) 3-oxo-C_12_-HSL in the ligand-binding cavity of LasR in the closed-loop conformation. **Right panel**: close-up view of the superposed structures of native (gold) and predicted (silver) compound **19** in the ligand-binding cavity of LasR in the open-loop conformation. Images were prepared and rendered using the PyMOL Molecular Graphics System, version 1.8 (Schrödinger LLC, Portland, OR, USA).

It has been shown experimentally that compound **19** binds the receptor when the so-called L3 loop (residues 40–51) points “outward” toward bulk solvent [44] and that 3-oxo-C12-HSL binds the receptor when this loop exists in an “inward” conformation [45]. A root-mean-square (r.m.s.) deviation cut-off of 2 Å often serves as a criterion of the accuracy of redocking calculations [46]. Our pair-fitting analyses revealed that CB-Dock was reliably successful in generating the near-native poses of 3-oxo-C12-HSL and compound **19**, with r.m.s. deviations of 0.705 Å (over 21 heavy-atom pairs) and 1.139 Å (over 25 heavy-atom pairs), respectively (Figure 6). Both ligands have previously been shown to establish direct hydrogen-bonding interactions with Tyr56, Trp60, Asp73, Thr75, and Ser129 [44,45]. Through its two aromatic rings, compound **19** can also form π–π stacking interactions with Tyr64, Trp88, and Phe101.

**Figure 7:**
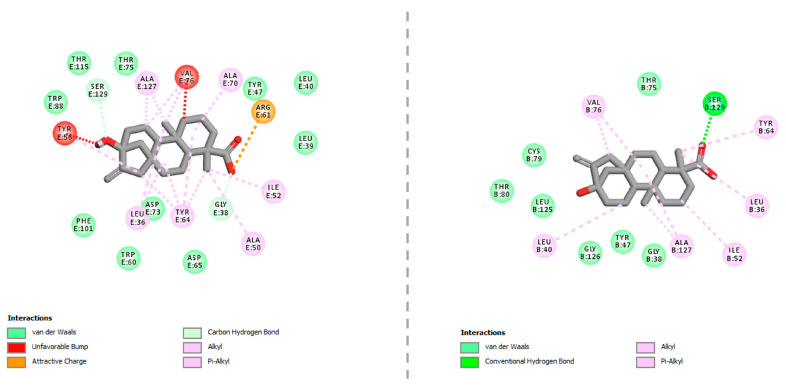
Favorable interactions between the LasR-LBD and steviol. **Left panel:** relative position of steviol with respect to the ligand-binding cavity of LasR in the closed-loop conformation. **Right panel:** relative position of steviol with respect to the ligand-binding cavity of LasR in the open-loop conformation. Images were prepared and rendered using Discovery Studio Visualizer, version 16.1.0 (Dassault Systèmes BIOVIA Corp., San Diego, CA, USA).

In cross-docking calculations, steviol was predicted to be the only active-site binder among the three natural sweeteners tested in the reporter assays. Interestingly, steviol showed favor to the comparatively large ligand-binding cavity of LasR in the open-loop conformation (Table 1). This could possibly be related to the size and geometry of the sweetener, as evidenced by the presence of steric bumps between steviol and Tyr56, Val76 from the ligand-binding cavity of LasR in the closed-loop conformation. Favorable non-covalent interactions between steviol and the LasR-LBD are demonstrated in Figure 7. Accordingly, steviol was able to engage in a hydrogen-bonding interaction with Ser129 (at 2.01 Å) through its carboxyl group. It also appeared to form π–alkyl or alkyl–alkyl type hydrophobic interactions with Leu36, Leu40, Ile52, Tyr64, Val76, and Ala127 (all within 5.50 Å) through its four-fused-ring system and methyl group. The position of steviol in the active-site cleft of LasR was further stabilized by van der Waals interactions with Gly38, Tyr47, Thr75, Cys79, Thr80, Leu125, and Gly126.

**Figure 8:**
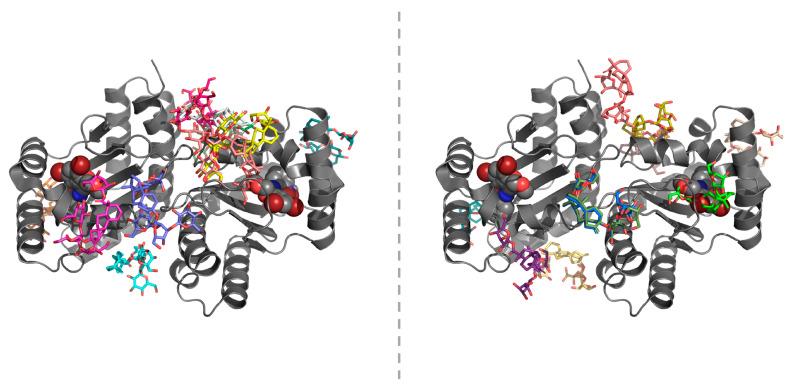
Representative (i.e., top-ranking) binding poses of the steviol glycosides from each cluster around the LasR LBD in the open-loop conformation. **Left panel:** Reb A. **Right panel:** stevioside. Images were prepared using the PyMOL Molecular Graphics System, version 1.8 (Schrödinger LLC, Portland, OR, USA).

In contrast to steviol, Reb A and stevioside could be found neither in the ligand-binding cavity of LasR in the closed-loop conformation nor in that of LasR open-loop conformation. This agrees with the fact that both Reb A and stevioside represent bulky steviol glycosides with relatively high molecular weights (967 g mol^−1^ and 805 g mol^−1^, respectively). Although these two sweeteners clustered at multiple sites other than the active site, no further attempt was made to explore potential binding sites for Reb A and stevioside on the LasR-LBD as the estimated binding energies of the top-ranking docking solutions from each cluster (10 in total) were very close to each other (−5.2 to −7.8 kcal mol^−1^ and −4.7 to −8.0 kcal mol^−1^ for closed-loop LasR-bound Reb A and stevioside, respectively; −5.8 to −7.9 kcal mol^−1^ and −6.1 to −7.7 kcal mol^−1^ for open-loop LasR-bound Reb A and stevioside, respectively). It is perhaps worth mentioning here that a number of the resulting docking solutions (particularly those located at the C-terminus of the LasR-LBD that is normally followed by the DNA-binding domain or DBD) are impractical under the conditions of physiological norm. Predicted binding poses of Reb A and stevioside around the LasR-LBD in the open-loop conformation are shown in Figure 8.

## 3. Discussion

In the gut, QS is pivotal in intra- and inter-bacterial communication, enabling many aspects of bacterial community behavior to be regulated. These bacteria use different signaling molecules as chemical messengers to relay information to each other. Many of these chemical messengers have been identified, however much remains unknown, including methods of disruption of the novel AHL signaling molecule 3-oxo-C12:2-HSL [36,37,38], resulting in possible dysbiosis. In the present work, we evaluated the effect of stevia extract, steviol glycosides (stevioside and Reb A), and their derived aglycon steviol on the AHL-dependent bacterial communication system.

We studied the effect of a CSHS on recombinant *E. coli* K802NR-pSB1075 reporter strain [40] using 3-oxo-C12-HSL, as 3-oxo-C12:2-HSL was unavailable commercially. In the absence of 3-oxo-C12-HSL, only residual light was produced by K802NR-pSB1075, which dramatically increased in the presence of 3-oxo-C12-HSL. The addition of different concentrations of CSHS in the presence of 3-oxo-C12-HSL caused a significant decrease in the bioluminescence emission of K802NR-pSB1075 relative to its control. CSHS showed no observable bactericidal effect. It appears that the impact of stevia on bacterial growth varies among strains. Studies have shown that stevioside and Reb A impair the growth of *Lactobacillus reuteri* strains [47] known for their protection of the epithelial barrier integrity [48]. Reb A had a bacteriostatic activity similar to those of saccharin, acesulfame K, and sucralose, although differing among *E. coli* strains [49].

Mammals cannot degrade steviol glycosides because they lack the necessary enzymes. This is done in the lower intestinal tract by gut microbiota that converts stevia glycosides into steviol [3,4,5,6]. While the gut microbial action on steviol glycosides has been extensively studied, the effect of such glycosides on the gut microbiota community ecosystem remains poorly understood. Concentration ranges for the various stevia compounds used in this study were chosen based on the published scientific studies for each of the compounds [8,16,17,47,49] and calculated to reflect concentration ranges within the acceptable daily intake (ADI) established by the Joint FAO/WHO Expert Committee on Food Additives (JECFA) [50]. Using pure samples of the stevia extract, glycosides (stevioside and Reb A), and their aglycon steviol, we showed that the agents have an inhibitory effect on our bacterial models. The pure stevia extract, stevioside, and steviol efficiently inhibit the K802NR-pSB1075 bioluminescence emission. The inhibitory effect of Reb A was not so effective on K802NR-pSB1075. As for PAO-JP2 (pKD-*rhlA*), all the stevia-derived components had efficient inhibitory action against the reporter strain’s bioluminescence emission. Our results, therefore, suggest that considerable consumption of stevia may disrupt the microbial balance.

Using a computational approach, we docked steviol, Reb A, and stevioside into the LasR-LBD in two different (loop-in and loop-out) conformations, whose 3D structures have been determined at atomistic resolution using X-ray crystallography. Our in-silico studies suggest a competitive-type inhibitory role for steviol, while Reb A and stevioside are likely to inhibit LasR-mediated QS in a non-competitive manner. Non-competitive antagonism has previously been suggested for LuxR by experimentation on several halogenated furanones that destabilize and facilitate the degradation of the receptor [51,52]. More recent research by Moore et al. [53] has provided further evidence of the existence of an allosteric QS-modulatory site on LasR (or on an interaction partner of LasR). Their observation has been verified by Paczkowski et al. [54], who demonstrated that flavonoids act as non-competitive inhibitors of QS by preventing LasR from binding to DNA. Intriguingly, the same researchers reported that flavonoids do not disrupt the formation of the LasR homodimer. Given the fact that Reb A and stevioside have many active rotatable bonds and that the AutoDock Vina scoring function has a standard error of approximately 3 kcal mol^−1^ [55], the presence of an alternative binding site on the LBD (if not on the DBD, or on the linker region between the LBD and the DBD) cannot be determined solely based on molecular docking. Therefore, biochemical and/or structural studies with full-length LasR are warranted before the proposed allosteric site of this transcriptional regulator can precisely be addressed.

In the current work, we did not attempt to model the RhlR protein, albeit its 40% similarity to the orphan LuxR-type protein SdiA from EHEC. We believe that docking the aforementioned natural sweeteners into a moderately reliable homology model of RhlR may not prove useful in this context, since it has previously been shown that even subtle conformational changes in the LBD lead to dramatic DBD motion can affect the biological function of LuxR-type transcriptional regulators [44,56]. Furthermore, it has been well established that LasR represents the master regulator of AHL signaling in *P. aeruginosa* and stimulates the production of the subordinate regulator RhlR [57,58]. Therefore, more research is needed to delineate whether the observed quorum-quenching activities of steviol, Reb A, and stevioside are mediated by direct RhlR antagonism or indirect (LasR-involved) RhlR antagonism in vivo.

Exposure to steviol glycosides or their derived aglycon steviol over time and at yet-to-be-determined quantities may negatively affect the gut microbial balance, by interfering in the AHL-mediated bacterial networking and thereby inducing associated health complications. Our assumption takes into consideration the fact that those with active inflammatory bowel disease (IBD), have shown significantly low 3-oxo-C12:2-HSL, corresponding to a significant decrease in one of the major phyla of the gut ecosystem, Firmicutes (particularly *C. coccoides*, *C. leptum,* and *F. prausnitzii*) [36]. Alteration in Firmicutes/Bacteroidetes ratio was shown to correlate with obesity [59,60,61], thus, such species alterations (taking note that 90% of the species in the gut belong to the Bacteroidetes and Firmicutes phyla [62]) may have far-reaching consequences. In another study, where suspensions of human feces were incubated with either stevioside or Reb A, a slight reduction was observed in the total count of anaerobic bacteria *Bacteroides* and *Lactobacilli* by stevioside, and in aerobic bacteria *Bifidobacteria* and *Enterococci* by Reb A [4]. The protective properties of 3-oxo-C12:2-HSL to enterocytes [37] suggest that the absence of the molecule in the event of an alteration on the communication network could render the enterocytes vulnerable.

With reference to the effects identified in our study and the growing consumption of stevia, we urge that more studies be conducted to help further elucidate the effects of these sweeteners and to adjust the highest daily doses recommended today.

## 4. Materials and Methods

### 4.1. Materials

Difco Luria-Bertani (LB) broth, Miller (10 g/L tryptone; 5 g/L yeast extract; 10 g/L NaCl) and Difco LB agar, Miller (10 g/L tryptone; 5 g/L yeast extract; 10 g/L NaCl; 15 g/L agar) obtained from Becton (Dickinson & Company, France). Trimethoprim, ampicillin, and C4-HSL were purchased from Sigma-Aldrich (St. Louis, MO, USA). Dimethyl sulfoxide (DMSO) was obtained from Fisher Scientific (Leics, UK). Commercial stevia supplement (CSS) was purchased from a local vendor. Stevia extract and stevioside were procured from HWI Pharma Services GmbH (Rülzheim, Germany). Reb A was purchased from PhytoLab (Vestenbergsgreuth, Germany). Due to the unavailability of commercial 3-oxo-C12:2-HSL (the QS molecule observed to be directly associated with normobiosis [36,37,38]), we used instead, the structurally similar molecule from *Pseudomonas aeruginosa* (3-oxo-C12-HSL), purchased from Sigma-Aldrich (St. Louis, MO, USA). Both show functional similarity by protecting the integrity of enterocytes [37,63].

### 4.2. Bacterial Strains

*Escherichia coli* K802NR strain containing a pSB1075 plasmid infused with a QS *lasI* promoter that regulates the transcription of the *luxCDABE* operon from *Vibrio fischeri* and the *lasRI* gene from *Pseudomonas aeruginosa* (obtained from Davies, J. University of British Columbia, Canada) were used. PAO-JP2 (pKD-*rhlA*), a *lasI-rhlI* double mutant of *P. aeruginosa* PAO1 harboring a pKD plasmid infused with the *rhlA* promoter coupled upstream to the *luxCDABE* box (obtained from Meijler M. M. Ben-Gurion University of the Negev, Be’er Sheva, Israel) was also used. All strain stocks were stored at −80 °C in 50% (*v*/*v*) of glycerol, a cryoprotectant additive.

### 4.3. Strain Cultivation

The K802NR-pSB1075 sensor strain was cultured on LB-agar plates (Difco Luria-Bertani medium, BD) containing 100 μg/mL ampicillin for 48 h at 37 °C in an incubator (Binder, Camarillo, CA, USA). Taking a single colony from the LB-agar plate, a starter culture was prepared by introducing the colony into a 10 mL LB medium containing 100 μg/mL ampicillin and grown overnight (with the cap of the tube half-open stabilized with autoclave tape) at 37 °C with shaking (at 140 rpm) on a rotary thermo-shaker (Gerhardt, Konigswinter, Germany). A 20 mL fresh LB medium, without antibiotic, was inoculated by 20 μL of the starter culture, and the fresh culture of bacteria was allowed to grow at 30 °C for about 4 h until the early log phase (OD_600_ ~0.2) without shaking. The PAO-JP2 (pKD-*rhlA*) strains were cultured on LB-agar plates (Difco Luria-Bertani medium, BD) containing 300 μg/mL trimethoprim for 24 h at 37 °C in the incubator (Binder, Camarillo, CA, USA). A single colony was introduced into 10 mL LB medium supplemented with 300 μg/mL trimethoprim (with the cap of the tube half-open stabilized with autoclave tape) and grown overnight at 37 °C with shaking (at 140 rpm) on a rotary thermo-shaker (Gerhardt, Konigswinter, Germany). The respective LB-agar plates containing K802NR-pSB1075 and PAO-JP2 (pKD-*rhlA*) strains were stored at 4 °C for future use, but not more than one month. The OD_600_ was determined by Ultrospec 2100 pro spectrophotometer (Amersham, Berks, UK).

### 4.4. Effects of Stevia Extracts and Stevia-Derived Compounds on Bacterial QS

The effect of stevia extracts on bacterial QS circuitry was determined at different concentrations, using the bioluminescence bioreporter assay. The bioluminescence of the K802NR-pSB1075 strain was measured in white opaque 96-well microtiter plates containing 10 μL of stevia extract or its pure components, 10 μL of 3-oxo-C_12_-HSL (final concentration 5 × 10^−10^ M), and 80 μL bacterial culture (at OD_600_ ~0.2). The positive control contained 80 μL of the bacterial culture, 10 μL 3-oxo-C_12_-HSL, and 10 μL LB broth, while the negative control contained 80 μL of the bacterial culture and 20 μL LB broth. The bioluminescence was measured at 490 nm maintained at 26 °C for 20 h at 10-min intervals after 10 s shaking using the Luminoskan Ascent Luminometer (Thermo Fisher Scientific, Waltham, MA, USA). The bioluminescence of PAO-JP2 (pKD-*rhlA*) strains was also measured in a white opaque 96-well microtiter plate containing 10 μL of different concentrations of the stevia extract or its pure components, 80 μL of the bacterial cultures (at OD_600_ = 0.015), and 10 μL of C4-HSL (final concentration 10 μM). Bioluminescence intensity values were expressed in relative light units (RLUs). Stevia extract and stevioside were dissolved in distilled water, while Reb A and steviol were dissolved in 17% (*v*/*v*) DMSO.

### 4.5. LC-MS Analysis of CSHS

Stevia supplement was separated using reverse-phase liquid chromatography (ACQUITY UPLC^®^HSS T3 1.8 µm 2.1 × 100 mm). Supplement was diluted 1:100 and 4 μL of the sample was injected. Fractions were eluted with an isocratic profile with a mobile phase of 35% acetonitrile in water with 0.1% formic acid for 5 min, followed by a gradient from 1% to 99% acetonitrile over 33 min, at a flow rate of 0.4 mL/min. For identification of stevia components, the LC fractions were subjected to electrospray ionization mass spectrometry (ESI-MS); Ion Trap MS Esquire 3000 Plus (Q Exactive, Thermo Scientific, Waltham, MA, USA), under positive-ion conditions [41]. In addition, fragmentation was performed with PRM-MS under positive and negative modes.

### 4.6. Molecular Docking

The three-dimensional (3D) structure of the homodimeric biological assembly (chains E and G) of the *P. aeruginosa* LasR ligand-binding domain (LBD) complexed with the native autoinducer 3-oxo-C_12_-HSL [45] (PDB entry: 2UV0; resolution: 1.80 Å; *R*-value, free: 0.254; *R*-value, work: 0.209) and the 3D structure of the homodimeric biological assembly (chains A and B) of the *P. aeruginosa* LasR LBD complexed with the non-native agonist compound **19** [44] (PDB entry: 6D6P; resolution: 1.65 Å; *R*-value, free: 0.248; *R*-value, work: 0.219) were downloaded from the RCSB Protein Data Bank [64] (available at https://www.rcsb.org/). The atomic coordinates of the two proteins were used as input to the Dock Prep tool of UCSF Chimera, version 1.11.2 [65], by which the receptors were prepared according to the following scheme: (i) solvent was deleted; (ii) selenomethionine residues were mutated into methionine residues (only applicable to the PDB entry 2UV0); (iii) hydrogen atoms were added (considering hydrogen-bonding interactions); (iv) ligand atoms/bonds were deleted. The isomeric SMILES strings of the natural sweeteners steviol (PubChem entry: 40619124), Reb A (PubChem entry: 6918840), and stevioside (PubChem entry: 442089) were retrieved from the PubChem Compound database [66] (available at https://pubchem.ncbi.nlm.nih.gov/) and submitted to an online 3D structure generator maintained by Biomodeling Research Co. Ltd. (Shanghai, China) (available at https://demo1.biomodeling.co.jp/) to translate them into MOL2 files using the myPresto programs Hgene, tplgeneL, cosgene, and tpl2mol2. The binding poses of the natural sweeteners of interest were predicted using a cavity detection-guided, AutoDock Vina-based blind docking algorithm adopted by the CB-Dock webserver [67] (available at http://cao.labshare.cn/cb-dock/). The number of cavities for docking was set from 5 to 10 for extra precision. The resulting docking solutions were prioritized based on their estimated binding energies, and favorable non-covalent interactions between the LasR LBDs and the selected natural sweeteners were visualized using Discovery Studio Visualizer, version 16.1.0 (Dassault Systèmes BIOVIA Corp., San Diego, CA, USA).

### 4.7. Statistical Analysis

Statistical analysis of data was performed using GraphPad Prism Software version 6.00 for Windows (La Jolla, CA, USA). All tests were compared with the controls and the *p*-values were calculated by Student’s *t*-test. Each data point on the graphs represents an average of three different experimental readings, expressed as mean ± standard deviation (SD), to ensure the reproducibility of the results.

## Figures and Tables

**Table 1:** Energetic parameters (docking scores) associated with the binding of steviol at the active site of the LasR-LBD.

	Closed-Loop Conformation (PDB Entry: 2UV0)		Open-Loop Conformation (PDB Entry: 6D6P)	
	Chain E	Chain G	Chain A	Chain B
Steviol	−3.0 kcal mol^−1^	−2.5 kcal mol^−1^	−9.0 kcal mol^−1^	−8.7 kcal mol^−1^
3-Oxo-C_12_-HSL	−8.9 kcal mol^−1^	−8.9 kcal mol^−1^	−	−
Compound 19	−	−	−11.5 kcal mol^−1^	−10.9 kcal mol^−1^

## Supplementary Materials

The following are available, Figure S1: LC-MS chromatogram of the CSHS in parallel reaction monitoring (PRM) mode, Figure S2: Product ion spectra obtained from MS/MS (PRM) fragmentations in positive ion mode of Stevioside (C38H60O18 + H+. MS2 of 805.3853 m/z) in CSHS, Figure S3: Product ion spectra obtained from MS/MS (PRM) fragmentations in positive ion mode of Reb A. (C44H70O23 + H+. MS2 of 967.4380 m/z) in CSHS, Figure S4: Product ion spectra obtained from MS/MS (PRM) fragmentations in positive ion mode of Steviol (C_20_H_30_O_3_ + H^+^. MS2 of 319.2268 m/z) in CSHS.

## References

[1] Samuel P., Ayoob T.K., Magnuson A.B., Wölwer-Rieck U., Jeppesen B.P., Rogers J.P., Rowland I., Mathews R. (2018). Stevia Leaf to Stevia Sweetener: Exploring ItsScience, Benefits, and Future Potential. J. Nutr..

[2] Brandle J.E., Starratt A., Gijzen M. (1998). Stevia rebaudiana: Its agricultural, biological, and chemical properties. Can. J. Plant Sci..

[3] Hutapea A.M., Toskulkao C., Buddhasukh D., Wilairat P., Glinsukon T. (1997). Digestion of Stevioside, a Natural Sweetener, by Various Digestive Enzymes. J. Clin. Biochem. Nutr..

[4] Gardana C., Simonetti P., Canzi E., Zanchi R., Pietta P. (2003). Metabolism of Stevioside and Rebaudioside A from Stevia rebaudiana Extracts by Human Microflora. J. Agric. Food Chem..

[5] Nikiforov A.I., Rihner M.O., Eapen A.K., Thomas J.A. (2013). Metabolism and Toxicity Studies Supporting the Safety of Rebaudioside D. Int. J. Toxicol..

[6] Purkayastha S., Markosyan A., Prakash I., Bhusari S., Pugh G., Lynch B., Roberts A. (2016). Steviol glycosides in purified stevia leaf extract sharing the same metabolic fate. Regul. Toxicol. Pharmacol..

[7] Chen T.H., Chen S.C., Chan P., Chu Y.L., Yang H.Y., Cheng J.T. (2005). Mechanism of the hypoglycemic effect of stevioside, a glycoside of Stevia rebaudiana. Planta Med..

[8] Ilić V., Vukmirović S., Stilinović N., Čapo I., Arsenović M., Milijašević B. (2017). Insight into anti-diabetic effect of low dose of stevioside. Biomed. Pharmacother..

[9] Ripken D., van der Wielen N., Wortelboer H.M., Meijerink J., Witkamp R.F., Hendriks H.F.J. (2014). Steviol Glycoside Rebaudioside A Induces Glucagon-like Peptide-1 and Peptide YY Release in a Porcine ex Vivo Intestinal Model. J. Agric. Food Chem..

[10] van der Wielen N., ten Klooster J.P., Muckenschnabl S., Pieters R., Hendriks H.F.J., Witkamp R.F., Meijerink J. (2016). The Noncaloric Sweetener Rebaudioside A Stimulates Glucagon-Like Peptide 1 Release and Increases Enteroendocrine Cell Numbers in 2-Dimensional Mouse Organoids Derived from Different Locations of the Intestine. J. Nutr..

[11] Jeppesen P.B., Gregersen S., Poulsen C.R. (2000). Stevioside acts directly on pancreatic beta cells to secrete insulin: Actions independent of cyclic adenosine monophosphate and adenosine triphosphate-sensitive K^+^-channel activity. Metabolism.

[12] Philippaert K., Pironet A., Mesuere M., Sones W., Vermeiren L., Kerselaers S., Pinto S., Segal A., Antoine N., Gysemans C. (2017). Steviol glycosides enhance pancreatic beta-cell function and taste sensation by potentiation of TRPM5 channel activity. Nat. Commun..

[13] Becker S.L., Chiang E., Plantinga A., Carey H.V., Suen G., Swoap S.J. (2020). Effect of stevia on the gut microbiota and glucose tolerance in a murine model of diet-induced obesity. FEMS Microbiol. Ecol..

[14] Nettleton J.E., Cho N.A., Klancic T., Nicolucci A.C., Shearer J., Borgland S.L., Johnston L.A., Ramay R.H., Tuplin E.N., Chleilat F. (2020). Maternal low-dose aspartame and stevia consumption with an obesogenic diet alters metabolism, gut microbiota and mesolimbic reward system in rat dams and their offspring. Gut.

[15] Nettleton J.E., Klancic T., Schick A., Choo A.C., Shearer J., Borgland S.L., Chleilat F., Mayengbam S., Reimer R.A. (2019). Low-Dose Stevia (Rebaudioside A) Consumption Perturbs Gut Microbiota and the Mesolimbic Dopamine Reward System. Nutrients.

[16] Li S., Chen T., Dong S., Xiong Y., Wei H., Xu F. (2014). The Effects of Rebaudioside A on Microbial Diversity in Mouse Intestine. Food Sci. Technol. Res..

[17] Mahalak K., Firrman J., Tomasula P.M., Nunez A., Lee J.-J., Bittinger K., Rinaldi M., Liu L. (2020). The impact of steviol glycosides and erythritol on the human and Cebus apella gut microbiome. J. Agric. Food Chem..

[18] Jimenez A.G., Sperandio V., Tommonaro G. (2019). Quorum Sensing and the Gut Microbiome. Quorum Sensing: Molecular Mechanism and Biotechnological Application.

[19] Atkinson S., Williams P. (2009). Quorum sensing and social networking in the microbial world. J. R. Soc. Interface.

[20] Mukherjee S., Bassler B.L. (2019). Bacterial quorum sensing in complex and dynamically changing environments. Nat. Rev. Microbiol..

[21] Azimi S., Klementiev A.D., Whiteley M., Diggle S.P. (2020). Bacterial Quorum Sensing During Infection. Annu. Rev. Microbiol..

[22] Wang S., Payne G.F., Bentley W.E. (2020). Quorum Sensing Communication: Molecularly Connecting Cells, Their Neighbors, and Even Devices. Annu. Rev. Chem. Biomol. Eng..

[23] Bassler L.B., Greenberg P.E., Stevens M.A. (1997). Cross-Species Induction of Luminescence in the Quorum-Sensing Bacterium Vibrio harveyi. J. Bacteriol..

[24] Chen X., Schauder S., Potier N., Van Dorsselaer A., Pelczer I., Bassler B.L., Hughson F.M. (2002). Structural identification of a bacterial quorum-sensing signal containing boron. Nature.

[25] Xavier K.B., Bassler B.L. (2005). Interference with AI-2-mediated bacterial cell–cell communication. Nature.

[26] An J.H., Goo E., Kim H., Seo Y.-S., Hwang I. (2014). Bacterial quorum sensing and metabolic slowing in a cooperative population. Proc. Natl. Acad. Sci. USA.

[27] Zhao X., Liu X., Xu X., Fu V.Y. (2017). Microbe social skill: The cell-to-cell communication between microorganisms. Sci. Bull..

[28] Lazazzera B.A. (2001). The intracellular function of extracellular signaling peptides. Peptides.

[29] Okada M., Sato I., Cho S.J., Iwata H., Nishio T., Dubnau D., Sakagami Y. (2005). Structure of the Bacillus subtilis quorum-sensing peptide pheromone ComX. Nat. Chem. Biol..

[30] Thoendel M., Kavanaugh J.S., Flack C.E., Horswill A.R. (2011). Peptide Signaling in the Staphylococci. Chem. Rev..

[31] Whitehead N.A., Barnard A.M.L., Slater H., Simpson N.J.L., Salmond G.P.C. (2001). Quorum-sensing in Gram-negative bacteria. FEMS Microbiol. Rev..

[32] Dobretsov S., Teplitski M., Paul V. (2009). Mini review: Quorum sensing in the marine environment and its relationship to biofouling. Biofouling.

[33] Dong Y.-H., Wang L.-H., Xu J.-L., Zhang H.-B., Zhang X.-F., Zhang L.-H. (2001). Quenching quorum-sensing-dependent bacterial infection by an N-acyl homoserine lactonase. Nature.

[34] Sitnikov D.M., Schineller J.B., Baldwin T.O. (1995). Transcriptional regulation of bioluminesence genes from Vibrio fischeri. Mol. Microbiol..

[35] Waters C.M., Bassler B.L. (2005). Quorum sensing: Cell-to-cell communication in bacteria. Annu. Rev. Cell Dev. Biol..

[36] Landman C., Besse A., Maubert M., Brot L., Humbert L., Cosnes J., Beaugerie L., Trugnan G., Sokol H., Rainteau D. (2013). Quorum Sensing Driven by *N*-Acyl-Homoserine Lactone in Inflammatory Bowel Diseases Associated Dysbiosis. Gastroenterology.

[37] Le Balc’h E., Landman C., Tauziet E., Brot L., Quevrain E., Rainteau D., Grill J.-P., Thenet S., Seksik P. (2017). 3-oxo-C12:2-HSL, a new N-acyl-homoserine lactone identified in gut ecosystem exerts an anti-inflammatory effect and does not modify paracellular permeability. J. Crohns Colitis.

[38] Landman C., Grill J.-P., Mallet J.-M., Marteau P., Humbert L., Le Balc’h E., Maubert M.-A., Perez K., Chaara W., Brot L. (2018). Inter-kingdom effect on epithelial cells of the N-Acyl homoserine lactone 3-oxo-C12:2, a major quorum-sensing molecule from gut microbiota. PLoS ONE.

[39] Kim C.S., Gatsios A., Cuesta S., Lam Y.C., Wei Z., Chen H., Russell R.M., Shine E.E., Wang R., Wyche T.P. (2020). Characterization of Autoinducer-3 Structure and Biosynthesis in *E. coli*. ACS Cent. Sci..

[40] Goh E.B., Yim G., Tsui W., McClure J., Surette M.G., Davies J. (2002). Transcriptional modulation of bacterial gene expression by sub-inhibitory concentrations of antibiotics. Proc. Natl. Acad. Sci. USA.

[41] Steckel A., Schlosser G. (2019). An Organic Chemist’s Guide to Electrospray Mass Spectrometric Structure Elucidation. Molecules.

[42] Bukelman O., Amara N., Mashiach R., Krief P., Meijler M.M., Alfonta L. (2009). Electrochemical Studies of Biofilm Formation and Inhibition. Chem. Commun..

[43] Duan K., Surette M.G. (2007). Environmental Regulation of Pseudomonas aeruginosa PAO1 Las and Rhl Quorum-Sensing Systems. J. Bacteriol..

[44] O’Reilly M.C., Dong S.H., Rossi F.M., Karlen K.M., Kumar R.S., Nair S.K., Blackwell H.E. (2018). Structural and Biochemical Studies of Non-native Agonists of the LasR Quorum-Sensing Receptor Reveal an L3 Loop “Out” Conformation for LasR. Cell Chem. Biol..

[45] Bottomley M.J., Muraglia E., Bazzo R., Carfì A. (2007). Molecular insights into quorum sensing in the human pathogen Pseudomonas aeruginosa from the structure of the virulence regulator LasR bound to its autoinducer. J. Biol. Chem..

[46] Bursulaya B.D., Totrov M., Abagyan R., Brooks C.L. (2003). Comparative study of several algorithms for flexible ligand docking. J. Comput. Aided Mol. Des..

[47] Deniņa I., Semjonovs P., Fomina A., Treimane R., Linde R. (2013). The influence of stevia glycosides on the growth of *Lactobacillus reuteri* strains. Lett. Appl. Microbiol..

[48] Karimi S., Jonsson H., Lundh T., Roos S. (2018). *Lactobacillus reuteri* strains protect epithelial barrier integrity of IPEC-J2 monolayers from the detrimental effect of enterotoxigenic Escherichia coli. Physiol. Rep..

[49] Wang Q.-P., Browman D., Herzog H., Neely G.G. (2018). Non-nutritive sweeteners possess a bacteriostatic effect and alter gut microbiota in mice. PLoS ONE.

[50] Joint FAO/WHO expert committee on food additives. Proceedings of the Eighty-Seventh Meeting.

[51] Manefield M., Rasmussen T.B., Henzter M., Andersen J.B., Steinberg P., Kjelleberg S., Givskov M. (2002). Halogenated furanones inhibit quorum sensing through accelerated LuxR turnover. Microbiology.

[52] Koch B., Liljefors T., Persson T., Nielsen J., Kjelleberg S., Givskov M. (2005). The LuxR receptor: The sites of interaction with quorum-sensing signals and inhibitors. Microbiology.

[53] Moore J.D., Rossi F.M., Welsh M.A., Nyffeler K.E., Blackwell H.E. (2015). A Comparative Analysis of Synthetic Quorum Sensing Modulators in Pseudomonas aeruginosa: New Insights into Mechanism, Active Efflux Susceptibility, Phenotypic Response, and Next-Generation Ligand Design. J. Am. Chem. Soc..

[54] Paczkowski J.E., Mukherjee S., McCready A.R., Cong J.P., Aquino C.J., Kim H., Henke B.R., Smith C.D., Bassler B.L. (2017). Flavonoids Suppress Pseudomonas aeruginosa Virulence through Allosteric Inhibition of Quorum-sensing Receptors. J Biol. Chem..

[55] Trott O., Olson A.J. (2010). AutoDock Vina: Improving the speed and accuracy of docking with a new scoring function, efficient optimization, and multithreading. J. Comput. Chem..

[56] Chen G., Swem L.R., Swem D.L., Stauff D.L., O’Loughlin C.T., Jeffrey P.D., Bassler B.L., Hughson F.M. (2011). A strategy for antagonizing quorum sensing. Mol. Cell.

[57] Pessi G., Haas D. (2000). Transcriptional control of the hydrogen cyanide biosynthetic genes *hcnABC* by the anaerobic regulator ANR and the quorum-sensing regulators LasR and RhlR in Pseudomonas aeruginosa. J. Bacteriol..

[58] Gilbert K.B., Kim T.H., Gupta R., Greenberg E.P., Schuster M. (2009). Global position analysis of the Pseudomonas aeruginosa quorum-sensing transcription factor LasR. Mol. Microbiol..

[59] Ley R., Turnbaugh P., Klein S., Gordon J.I. (2006). Human gut microbes associated with obesity. Nature.

[60] Koliada A., Syzenko G., Moseiko V., Budovska L., Puchkov K., Perederiy V., Gavalko Y., Dorofeyev A., Romanenko M., Tkach S. (2017). Association between body mass index and Firmicutes/Bacteroidetes ratio in an adult Ukrainian population. BMC Microbiol..

[61] Riva A., Borgo F., Lassandro C., Verduci E., Morace G., Borghi E., Berry D. (2017). Pediatric obesity is associated with an altered gut microbiota and discordant shifts in Firmicutes populations. Environ. Microbiol..

[62] Lane E.R., Zisman T., Suskind D. (2017). The microbiota in inflammatory bowel disease: Current and therapeutic insights. J. Inflamm. Res..

[63] Landman C., Clement M., Nsiri H., Quevrain E., Bazin T., Brot L., Grill J.-P., Maubert M.-A., Humbert L., Sokol H. (2015). The N- Acyl-Homoserine Lactone 3-oxo-C12, an Inter-Bacterial Signaling Molecule (Involved in Quorum Sensing), Exerts Effects on the Host: Thus, Implicating Quorum Sensing in Inflammatory Bowel Disease. J. Crohns Colitis.

[64] Berman H.M., Westbrook J., Feng Z., Gilliland G., Bhat T.N., Weissig H., Shindyalov I.N., Bourne P.E. (2000). The Protein Data Bank. Nucleic Acids Res..

[65] Pettersen E.F., Goddard T.D., Huang C.C., Couch G.S., Greenblatt D.M., Meng E.C., Ferrin T.E. (2004). UCSF Chimera—A visualization system for exploratory research and analysis. J. Comput. Chem..

[66] Kim S., Thiessen P.A., Bolton E.E., Chen J., Fu G., Gindulyte A., Han L., He J., He S., Shoemaker B.A. (2016). PubChem Substance and Compound databases. Nucleic Acids Res..

[67] Liu Y., Grimm M., Dai W.T., Hou M.C., Xiao Z.X., Cao Y. (2020). CB-Dock: A web server for cavity detection-guided protein-ligand blind docking. Acta Pharmacol. Sin..

