# Supplementary material for: Anti-Quorum Sensing Activity of Stevia Extract, Stevioside, Rebaudioside A and Their Aglycon Steviol
Source: Molecules. 2020 Nov 23;25(22):5480. doi: 10.3390/molecules25225480 (PMC7700441; doi:10.3390/molecules25225480)
Supplement: Supplementary file 1 [file molecules-25-05480-s001.pdf]

Supplementary Materials:

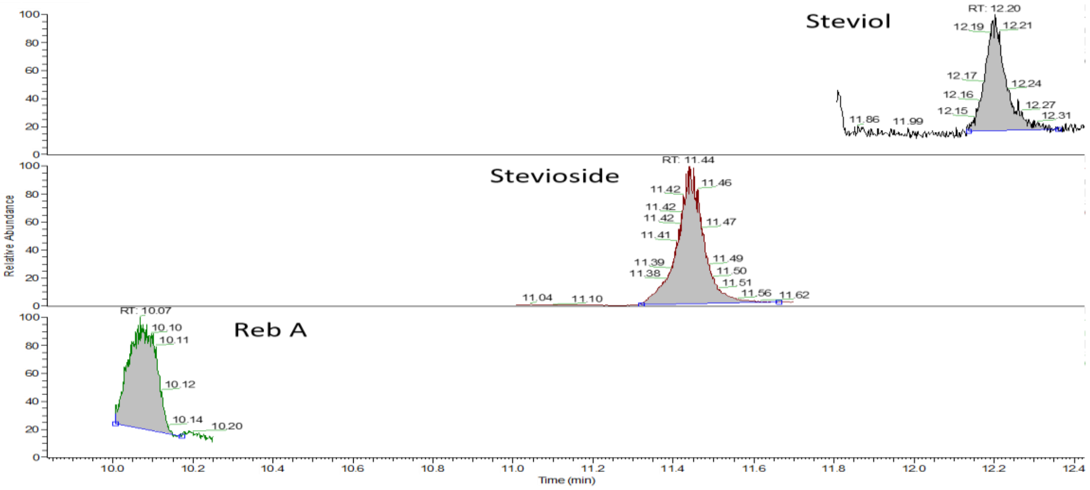

Figure S1. LC-MS chromatogram of the CSHS in parallel reaction monitoring (PRM) mode.

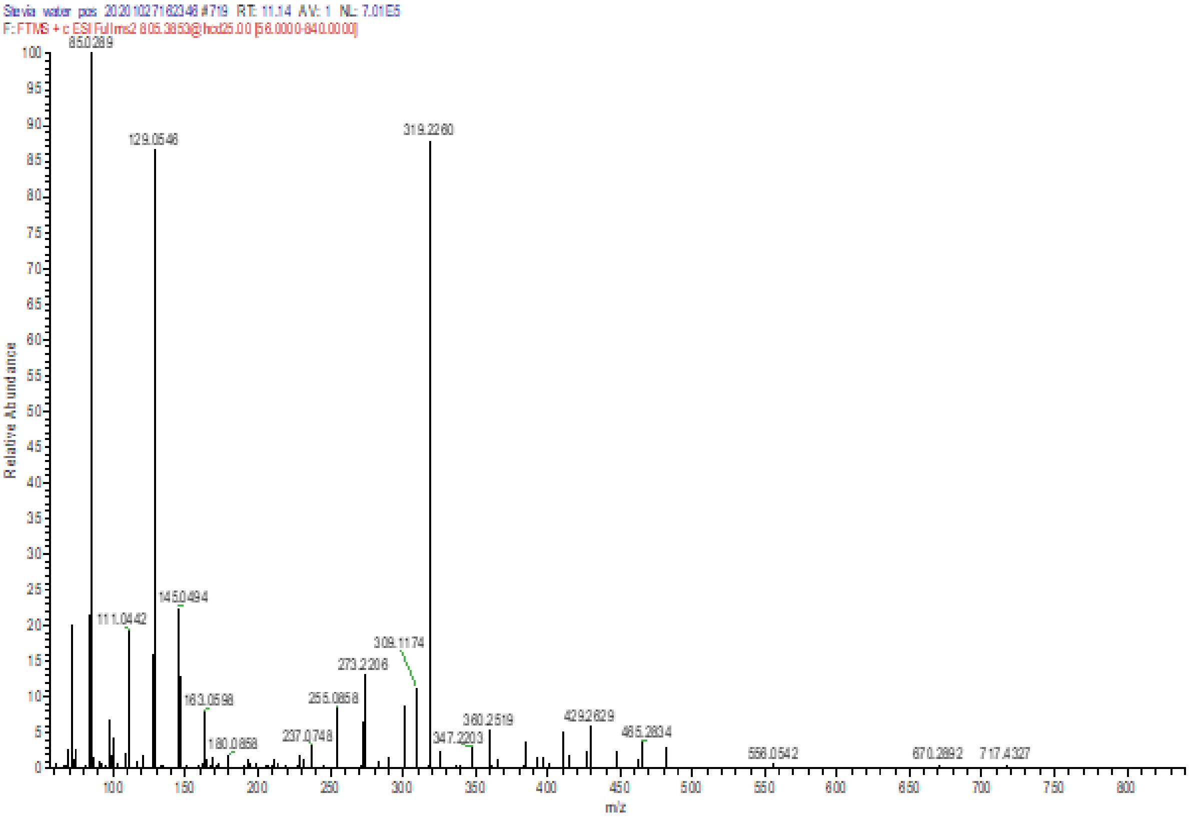

Figure S2: Product ion spectra obtained from MS/MS (PRM) fragmentations in positive ion mode of Stevioside ($C_{38}H_{60}O_{18} + H^{+}$. MS2 of 805.3853 *m/z*) in CSHS.

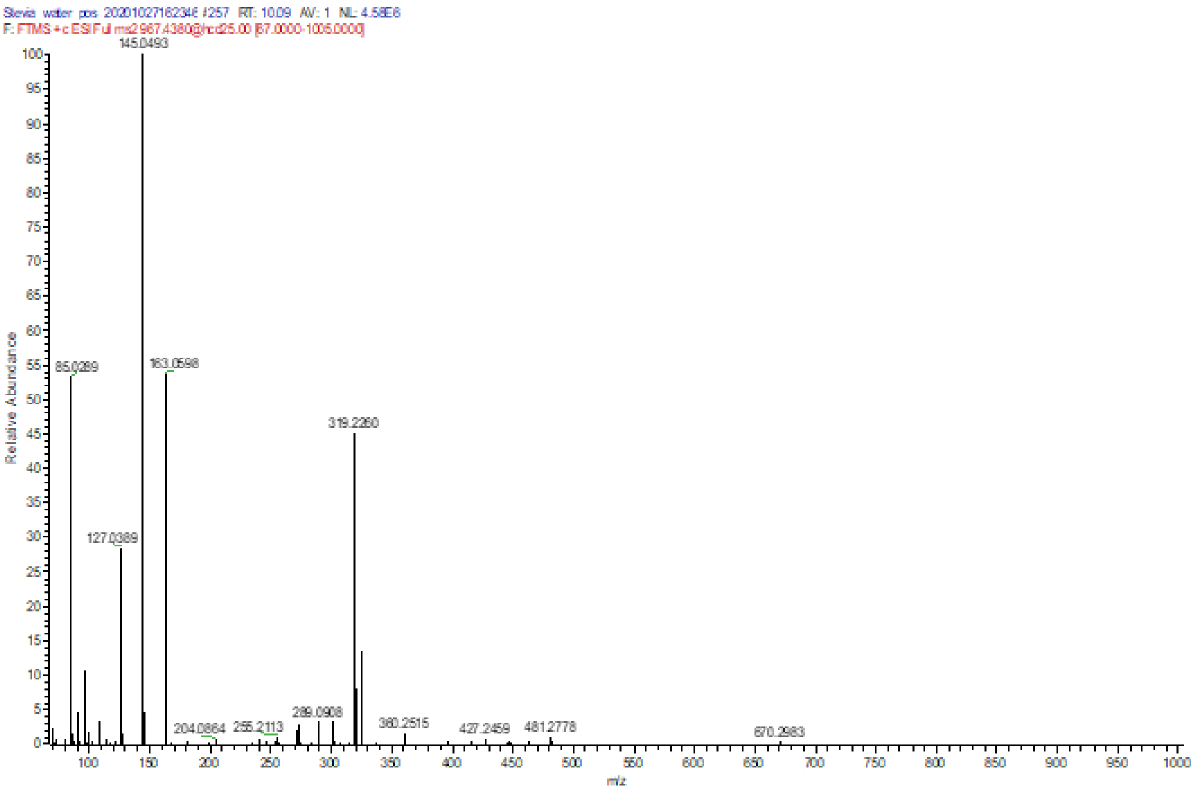

Figure S3: Product ion spectra obtained from MS/MS (PRM) fragmentations in positive ion mode of Reb A. ($C_{44}H_{70}O_{23} + H^{+}$. MS2 of 967.4380 *m/z*) in CSHS.

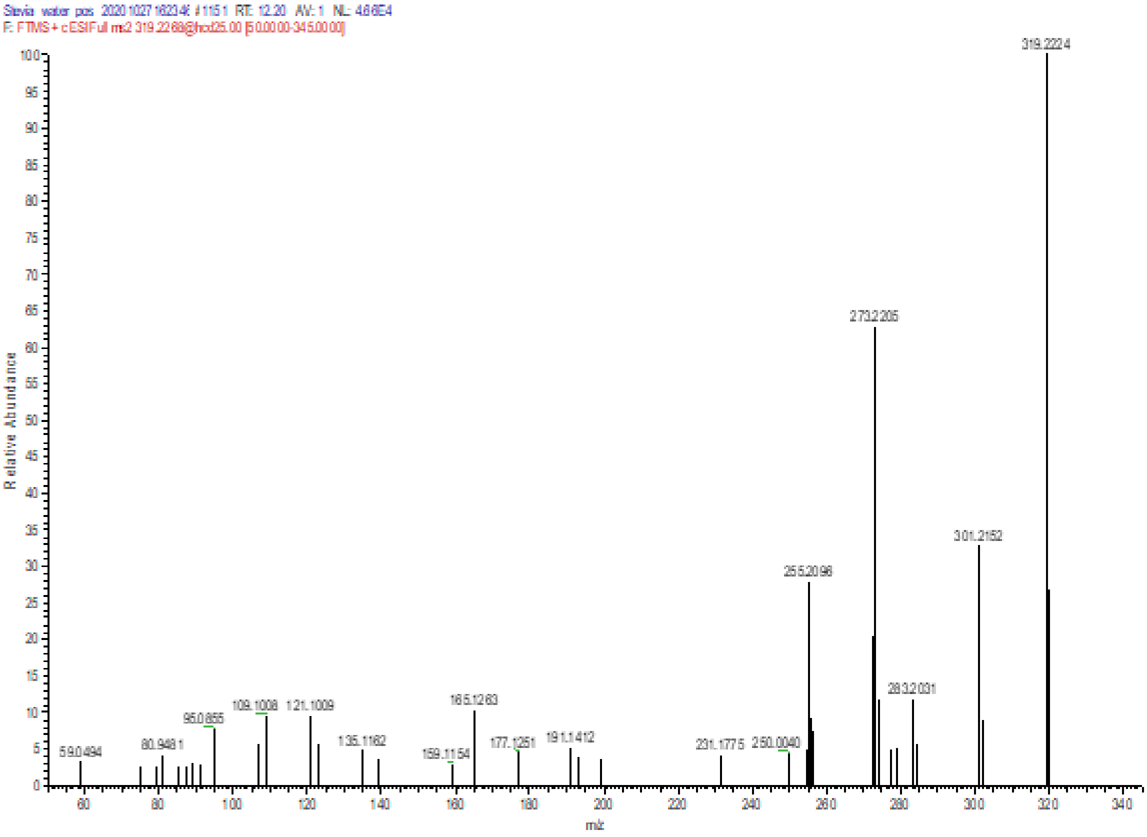

Figure S4: Product ion spectra obtained from MS/MS (PRM) fragmentations in positive ion mode of Steviol ($C_{20}H_{30}O_{3} + H^{+}$. MS2 of 319.2268 *m/z*) in CSHS.
